# A directional 3D neurite outgrowth model for studying motor axon biology and disease

**DOI:** 10.1038/s41598-021-81335-z

**Published:** 2021-01-22

**Authors:** Xandor M. Spijkers, Svetlana Pasteuning-Vuhman, Jennifa C. Dorleijn, Paul Vulto, Nienke R. Wevers, R. Jeroen Pasterkamp

**Affiliations:** 1 MIMETAS BV, Organ-On-a-Chip Company, 2333 CH Leiden, The Netherlands; 2 Department of Translational Neuroscience, University Medical Center Utrecht Brain Center, Utrecht University, 3584 CG Utrecht, The Netherlands; 3 Department of Cell and Chemical Biology, Leiden University Medical Centre, Einthovenweg 20, 2333 ZC Leiden, The Netherlands

**Keywords:** Cellular neuroscience, Motor neuron disease

## Abstract

We report a method to generate a 3D motor neuron model with segregated and directed axonal outgrowth. iPSC-derived motor neurons are cultured in extracellular matrix gel in a microfluidic platform. Neurons extend their axons into an adjacent layer of gel, whereas dendrites and soma remain predominantly in the somal compartment, as verified by immunofluorescent staining. Axonal outgrowth could be precisely quantified and was shown to respond to the chemotherapeutic drug vincristine in a highly reproducible dose-dependent manner. The model was shown susceptible to excitotoxicity upon exposure with excess glutamate and showed formation of stress granules upon excess glutamate or sodium arsenite exposure, mimicking processes common in motor neuron diseases. Importantly, outgrowing axons could be attracted and repelled through a gradient of axonal guidance cues, such as semaphorins. The platform comprises 40 chips arranged underneath a microtiter plate providing both throughput and compatibility to standard laboratory equipment. The model will thus prove ideal for studying axonal biology and disease, drug discovery and regenerative medicine.

## Introduction

Motor neurons project their axons to innervate other neurons and smooth- and skeletal muscle, allowing muscles in the body to contract. Impaired motor neuron function due to nerve injury or disease, affects voluntary movement and can cause paralysis. Although peripheral nerves are able to regenerate after injury, complete recovery does not occur^1–3^. Disease is another cause of disrupted motor neuron and axon function. In amyotrophic lateral sclerosis (ALS), upper and lower motor neurons undergo progressive degeneration leading to muscle denervation, paralysis and death within 3–5 years after diagnosis^4,5^. Although exact causes are still unknown, aberrances in axonal stability, axonal transport, and axon growth dynamics have been reported amongst other hallmarks^5–10^. Accordingly, there is an increased research interest in studying axonal repair after peripheral nerve injury, as well as in studying axonal pathology in ALS. In vitro models that are relevant in their physiological behavior and allow spatially segregated interrogation of motor neuron somata and axons, are critical in advancing our understanding of peripheral nerve injury and disease, and in developing novel curing or regenerating therapies.

The introduction of induced pluripotent stem cell (iPSC) technology and subsequent differentiation into human motor neurons provides a unique opportunity to study motor neuron disease and nerve regeneration mechanisms. In particular, the generation of iPSC-derived motor neurons from patients carrying specific mutations is a powerful tool for in vitro disease modeling. Most studies have focused on iPSC-derived lower motor neurons to study ALS. Lower motor neurons from familial ALS patients carrying superoxide dismutase 1 (*SOD1*) mutations showed various disease phenotypes compared to healthy controls, including reduced survival, abnormal mitochondrial mobility and endoplasmic reticulum (ER) stress^11,12^. Lower motor neurons derived from patients carrying a mutation in the *fused in sarcoma* (*FUS*) gene showed cytoplasmic mislocalization of FUS and accumulation into SGs, in addition to aberrant axonal transport of mitochondria and ER vesicles^13,14^. Relevant disease phenotypes were also observed using iPSC technology for other common mutations in familial ALS, such as *chromosome 9 open reading frame 72* (*C9ORF72*) and *TAR DNA binding protein 43* (*TDP-43*) mutations. An extensive overview of this topic is provided elsewhere^15^.

Despite recent developments in iPSC technology, progress in dissecting ALS disease mechanisms, or in developing efficient therapies, is often hampered by a lack of relevant and scalable in vitro platforms that can rapidly be used to study axonal pathology. Two-dimensional culture platforms have been used extensively to model neurite outgrowth and regeneration, reviewed by Al-Ali et al.^16^. Low-density neuronal cultures allow for assessment of morphology and neurite outgrowth of individual cells. These cultures can be used to assess the effects of genetic mutations and effects of neurotoxic and neuroprotective compounds. Alternatively, stripe assays and spot assays can be employed. These 2D assays make use of control substances and test substances applied in stripes or spots, after which neurons are plated and neurite outgrowth onto the stripes or into the spots is detected via microscopy^17,18^. High-density neuronal cultures do not allow for assessment of morphology and neurite outgrowth of single cells. However, they can still provide information on overall outgrowth in a system, e.g. via scratch assays, in which cells and neurites are removed or damaged mechanically and recovery can be observed by microscopy^19^.

While traditional 2D cultures such as the ones described above have shown invaluable for studying neurite outgrowth, it has been reported that neuronal cultures in 3D display increased survival rates and distinct gene expression profiles as compared to 2D monolayers^20^. More importantly, traditional cultures do not allow for directed neurite outgrowth and separation of cell somata and neurites, which is of major interest in studying motor axon biology. Microfluidic systems are emerging as novel tools that counterbalance important drawbacks of commonly used in vitro culture setups. Microfluidic systems provide excellent spatial control of cells and matrices^21^, and are routinely used to study axon outgrowth. Drawing inspiration from early work by Robert Campenot^22^, Taylor and colleagues developed a microfluidic chip that allowed assessment of neurite outgrowth in a two-dimensional setting by employing microgrooves^23^. A similar setup was employed by many others to generate compartmentalized neuronal cultures that segregate cell somata and axons^24–30^. Others have developed three-dimensional microfluidic cultures for the same purpose, showing compartmentalized somata and axon outgrowth and connection to skeletal muscle^31,32^. Although these platforms were successfully used to study axonal injury and regeneration, they are typically implemented as a single chip system and not compatible with automation, and therefore not suited for routine experimentation or high-throughput screening. In addition, most chips are fabricated only by the research groups that developed them, and use a silicon rubber as prototyping material^33^. This material typically leads to high absorption of hydrophobic compounds. Moreover, the fabrication of most chips is cumbersome and hampers routine adoption by other laboratories.

Here we report a method to generate a compartmentalized neurite outgrowth model in a 3D fashion, using a high-throughput microfluidic platform that is made out of materials that are biocompatible and non-absorbing^34^. iPSC-derived motor neuron progenitors were differentiated into cholinergic lower motor neurons in 3D in an extracellular matrix gel. While somata and dendrites stayed predominantly within the somal compartment, axons extended into an adjacent layer of gel, resulting in spatial segregation of axons from somata and dendrites. Axon outgrowth and stability were assessed in response to the chemotherapeutic drug vincristine, which is known to bind tubulin monomers and disrupt microtubule networks^35^. In addition, cultures were exposed to high concentrations of glutamate or to sodium arsenite, mimicking excitotoxicity and SG formation seen in ALS pathology. Finally, axon attraction and repulsion through gradients of chemotrophic molecules was studied, which is of interest for regenerative approaches. The OrganoPlate platform used comprises 40 chips underneath a microtiter plate, enabling both throughput and compatibility of the platform. We envision the use of this model in studying motor neuron disease- and regeneration-related processes that may aid the discovery of novel therapeutic directions.

## Results

### Culture of motor neuron progenitors in the OrganoPlate

**Figure 1:**
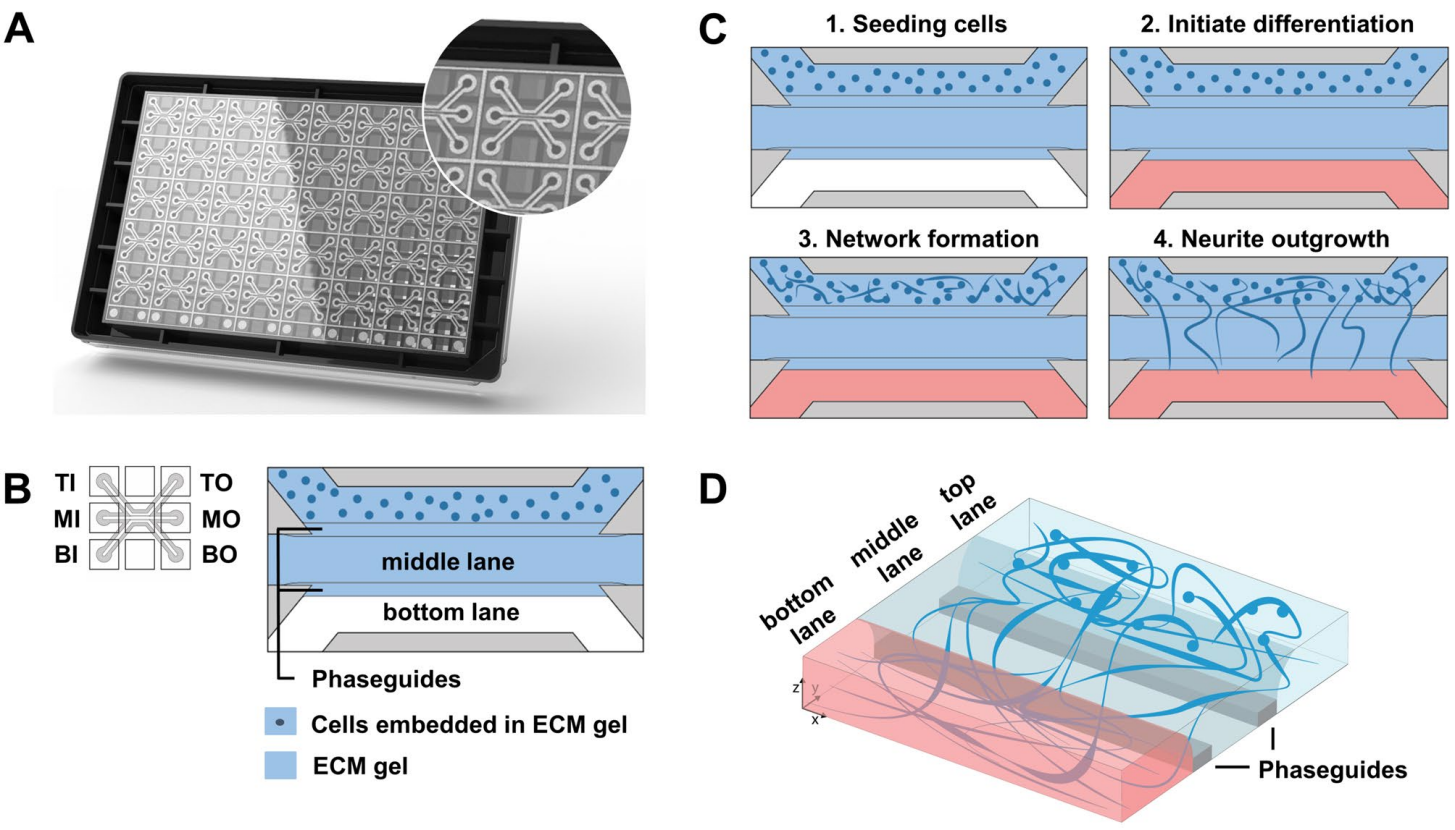
Motor neuron progenitors form 3D networks in the OrganoPlate. (**A**) Image of an OrganoPlate 3-lane. The OrganoPlate is a microfluidic platform based on a 384-well plate design containing 40 individual tissue culture chips. The image is copyright of MIMETAS BV. (**B**) Each tissue culture chip contains 3 microfluidic lanes that join in the center of the chip. The 3-lane architecture in the chips’ center is assured by phaseguides that enable patterning of extracellular matrix gel. Inlet and outlet wells allow access to each lane: top inlet (TI) and top outlet (TO) wells allow access to top lane; middle inlet (MI) and outlet (MO) wells to middle lane; and bottom inlet (BI) and outlet (BO) wells to the bottom lane. Matrigel-GFR without cells is added to the middle lane. Following gelation, motor neuron progenitors embedded in matrigel-GFR are seeded in the top lane. (**C**) Schematic overview of cell seeding procedure. Matrigel-GFR is seeded in the middle lane and MNPs embedded in matrigel-GFR are seeded in the top lane (panel 1). MNPs form networks in the top lane (panel 2), after which neurites protrude into the middle and eventually bottom lane (panel 3). Blue color indicates presence of gel, red color indicates presence of medium. (**D**) Schematic overview of the spatial segregation of motor neuron somata (top lane) and axons (middle and bottom lanes) in OrganoPlate 3-lane cultures.

The OrganoPlate 3-lane (Fig. 1A) is a microfluidic platform comprising 40 tissue culture chips which can be used for the generation of miniaturized tissues^34^. It employs a microtiter plate design, making it compatible with standard laboratory equipment. A schematic depiction of a single chip is shown in Fig. 1B. Within a chip, the microfluidic lanes are patterned by small pressure barriers termed phaseguides^36^. Phaseguides cause a change in capillary pressure subsequently preventing fluids to overflow into the adjacent lane, thereby patterning them without the use of artificial membranes. We leveraged the OrganoPlate 3-lane to generate a segregated motor neuron neurite outgrowth model in which developing neurites are separated from cell somata (Fig. 1B). Matrigel-growth factor reduced (GFR) was dispensed in the middle lane, after which motor neuron progenitors (MNPs) embedded in matrigel-GFR were added to the top lane. Motor neuron differentiation medium was then added to the top and bottom lane to differentiate the MNPs into mature motor neurons. In this set-up, MNP differentiation was accompanied by neurite extension into the adjacent microfluidic lane. A schematic overview of the procedure is shown in Fig. 1C,D.

### Characterization of motor neuron differentiation and neurite outgrowth in the OrganoPlate

**Figure 2:**
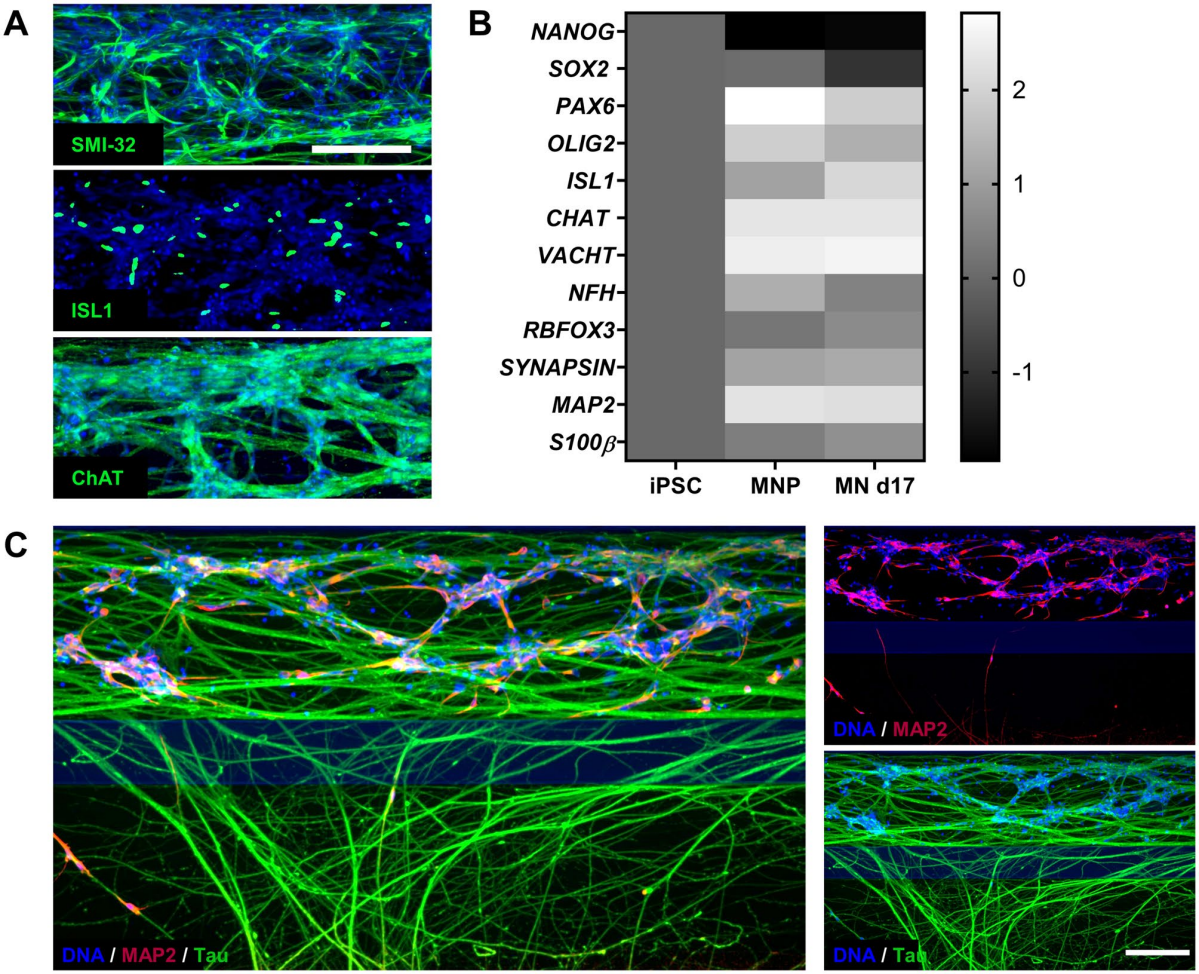
Progenitors differentiate into motor neurons and extend their axons into an adjacent gel lane. (**A**) MNPs were differentiated in the OrganoPlate for 17 days and immunostained for motor neuron markers SMI-32, ISL1, and CHAT. Maximum projection images of neuronal networks in the top lane are shown, the scale bar is 200 µm. (**B**) mRNA expression levels of induced pluripotent stem cells (iPSC), motor neuron progenitors (MNP) and motor neurons differentiated for 17 days (MN d17) in the OrganoPlate. Expression of markers of neural stem cells (*NANOG*, *SOX2*, *PAX6*), motor neuron progenitors (*OLIG2*), motor neurons (*ISL1*, *CHAT*, *RBFOX3*, *VACHT*, *NFH*), mature neurons (SYN1/*SYNAPSIN*, *MAP2*) and glial cells (*S100*β) was evaluated. Heatmap shows fold-change values as compared to expression in iPSCs, and the graphic representation was created using GraphPad Prism, version 8.3.1 (https://www.graphpad.com/scientific-software/prism/). (**C**) Motor neurons (day 17) were immunostained for dendritic marker MAP2 (red) and axonal marker TAU (green). Neurites protruding into the middle lane are mostly TAU-positive and MAP2-negative, indicating a predominantly axonal nature. Maximum projection images, the scale bar is 200 µm.

To characterize the cells in the OrganoPlate, we performed immunostaining and qPCR analyses. MNPs were differentiated in 3D in the OrganoPlate for 17 days and immunostained to confirm the presence of motor neuron markers SMI32, islet-1 (ISL1), and choline acetyltransferase (CHAT) (Fig. 2A). To evaluate the success of the differentiation, gene expression of the 17-day differentiated motor neurons was compared to non-differentiated MNPs and iPSCs. MNPs and differentiated motor neurons showed expression of motor neuron markers *ISL1*, *CHAT*, *NFH*, and *SLC18A3*, encoding the vesicular acetylcholine transporter (VACHT) protein, mostly at similar levels. This indicates that the MNPs used in this study already express many genes associated with motor neurons. However, the differentiated motor neurons showed reduced expression of progenitor markers *OLIG2*, *SOX2,* and *PAX6* compared to the MNPs, indicating further differentiation and maturation (Fig. 2B, Supplementary Fig. S1). The expression of the glial marker *S100β* was low and did not increase substantially over time. To characterize neurite outgrowth, cultures were immunostained for the somatodendritic marker microtubule-associated protein 2 (MAP2) and the axonal marker microtubule-associated protein TAU (MAPT)/TAU. Neurites penetrating the middle lane were mostly TAU-positive, while only some neurites contained MAP2 (Fig. 2C). These results indicated that the neurites protruding the adjacent lane were predominantly axonal.

Finally, we assessed spontaneous calcium fluctuations as an indication of electrophysiological activity. The calcium-sensitive dye Cal-520 AM was loaded into the cells, after which intracellular calcium fluctuations were detected using high-speed imaging (Supplementary Video S1). The videos show spontaneous calcium fluctuations confirming that the neurons are electrophysiologically active. Overall, these data show that differentiated motor neuron progenitors are mature, electrophysiologically active, and send their axons into the middle lane of the tissue culture chip.

### Neuronal network formation and quantification of axon outgrowth

**Figure 3:**
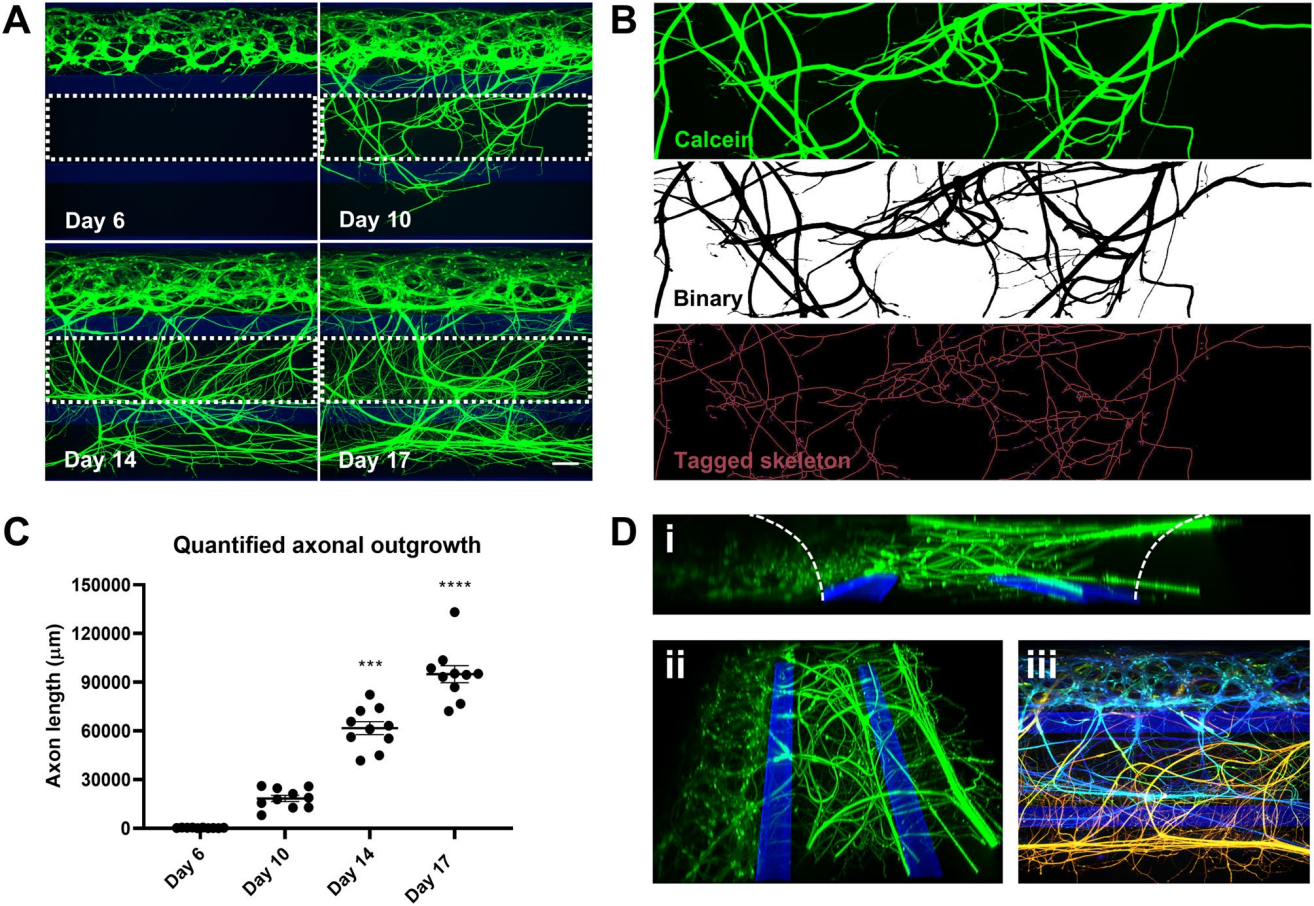
Characterization of 3D axon outgrowth. (**A**) Motor neuron axon outgrowth over time, visualized using calcein-AM dye. Dashed lines indicate region of interest (ROI) used for axon outgrowth quantification. Maximum projection images, the scale bar is 140 µm. (**B**) Quantification of axon outgrowth in the middle lane at day 17. Green-fluorescent signal of calcein-labeled axons was converted into a binary image, and subsequently converted into a skeletonized image that allows quantification of neurite length. (**C**) Quantification of motor neuron axon outgrowth over time using the procedure depicted in b. Graph shows mean neurite length ± SEM, n = 10, N = 2. *** (P < 0.001), **** (P < 0.0001); Kruskall–Wallis test. The graphic representation was created using GraphPad Prism, version 8.3.1 (https://www.graphpad.com/scientific-software/prism/). (**D**) 3D reconstruction of neuronal network and axonal outgrowth rendered from a confocal Z-stack, visualized using a calcein-AM dye at day 17. A side view is shown in panel i. Panel ii shows a top view. Panels i and ii were created using Imaris Viewer (https://imaris.oxinst.com/imaris-viewer). Panel iii depicts the Z-axis of the culture, in which structures at different depths within the chip are depicted using a color gradient to visualize the 3D nature of the culture. Deeper structures are depicted in blue, more superficial structures are depicted in green, followed by yellow and orange. Panel iii was created in ImageJ^76^.

Motor neurons send their axons over long distances to connect with their target cells, and changes in motor axon projections are a hallmark of motor neuron disease. Thus, as axon outgrowth and morphology are important parameters, we investigated our ability to quantify axon outgrowth in the OrganoPlate. MNPs seeded in the OrganoPlate formed neuronal networks in the top lane only during the first 6 days, after which they started to protrude their processes into the middle lane (Fig. 3A). This network became increasingly complex over time, where axons eventually penetrated into the bottom lane (Fig. 3A, Supplementary Fig. S2). Interestingly, the addition of 10 ng/mL brain-derived neurotrophic factor BDNF to the bottom lane increased cell migration into the middle lane (Supplementary Fig. S2). Moreover, upon filling the middle lane with a collagen-I ECM, axon outgrowth was substantially reduced (Supplementary Fig. S3). These observations indicate that specific molecular changes affect the behavior of motor neurons and axons in the OrganoPlate. To quantify the outgrowth, the raw signal from the middle lane (region of interest, ROI) was converted into a binary image where black signal indicates the presence of live cells and processes. The black signal was then skeletonized, after which total axon length was extracted (Fig. 3B). As expected, total axon outgrowth increased over time (Fig. 3C). Axon outgrowth was significantly increased at day 14 (P = 0.0003) and 17 (P < 0.0001) when compared to day 6. For situations of abundant outgrowth, an alternative quantification approach may be employed where black signal is quantified directly as a percentage of the total ROI measured (Supplementary Fig. S4). The first approach, in which axons are skeletonized, offers the advantage of quantifying axon length in micrometers. However, both quantification approaches yielded comparable results. To finally demonstrate the 3-dimensionality of the cultures, Z-slices from a single tissue culture chip were made and used for the generation of a 3D reconstruction (Fig. 3D, Supplementary Video S2). Taken together, these results show that motor neurons in the OrganoPlate increasingly extend their axons into adjacent lanes, which can be reliably quantified using different approaches.

### Robust modelling of disease-relevant phenotypes for screening

**Figure 4:**
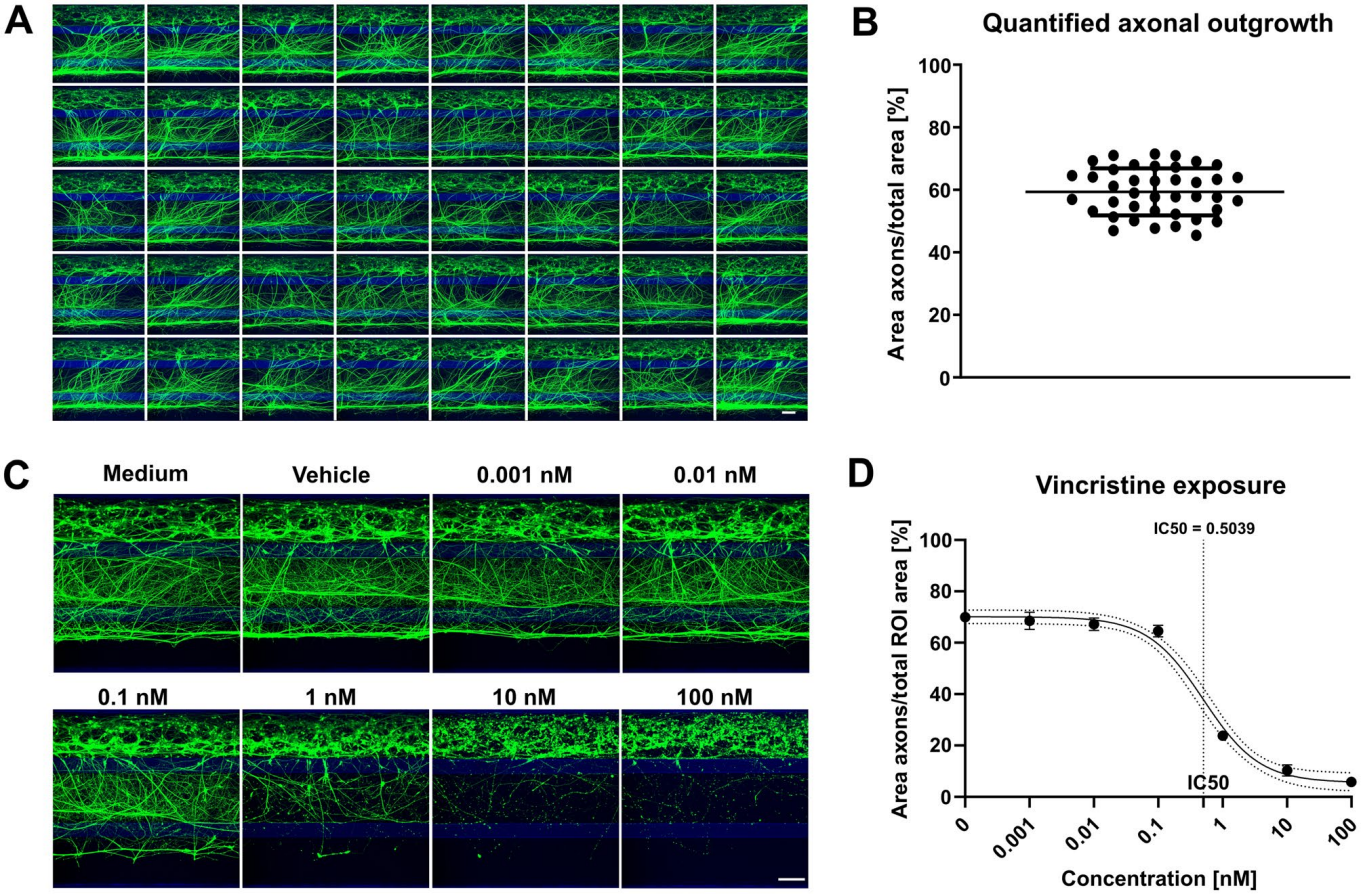
Robust 3D axonal outgrowth model allows assessment of neurite disrupting compounds. (**A**) Montage of a complete OrganoPlate containing 40 chips, each showing 3D axonal outgrowth at day 17 of culture visualized by calcein-AM dye. Maximum projection images, the scale bar is 200 µm. (**B**) Quantification of axonal outgrowth shown in (A), n = 40. Graph shows mean ± SD. (**C**) Motor neurons (day 17) were exposed to various concentrations of vincristine for 4 days. Cultures were visualized with calcein-AM dye. Maximum projection images, the scale bar is 200 µm. (**D**) Quantification of axonal outgrowth depicted in (**C**). Dose–response curve showing the effect of vincristine exposure on axonal outgrowth. Graph shows mean values ± SEM and a 95% confidence interval (dotted lines) around the curve. n = 5. The graphic representations in (**B**) and (**D**) were created using GraphPad Prism, version 8.3.1 (https://www.graphpad.com/scientific-software/prism/).

We next assessed the robustness of the outgrowth between chips and found highly similar outgrowth in 40 chips of a complete OrganoPlate 3-lane (Fig. 4A,B). As axon outgrowth was robust, we then proceeded with exposing our cultures to various concentrations of vincristine. Vincristine binds tubulin monomers and prevents their incorporation in the microtubule network, halting axon outgrowth and destabilizing developing axons. Vincristine concentrations of 0.01 nM and lower did not notably affect axon outgrowth, while concentrations of 10 nM and higher almost completely disrupted axons in the middle and bottom lane (Fig. 4C,D). In the OrganoPlate, vincristine is thus shown to have a dynamic range between 0.1 and 10 nM. In concordance, the IC50 of vincristine exposure was determined to be 0.5039 nM. To further characterize these experiments, LDH release was measured as an indicator of cytotoxicity. Vincristine substantially affected axon outgrowth, while only at the highest concentration tested vincristine may lead to increased LDH activity (P = 0.065; Supplementary Fig. S5). These results indicated that our treatment can specifically target axon outgrowth and stability.

**Figure 5:**
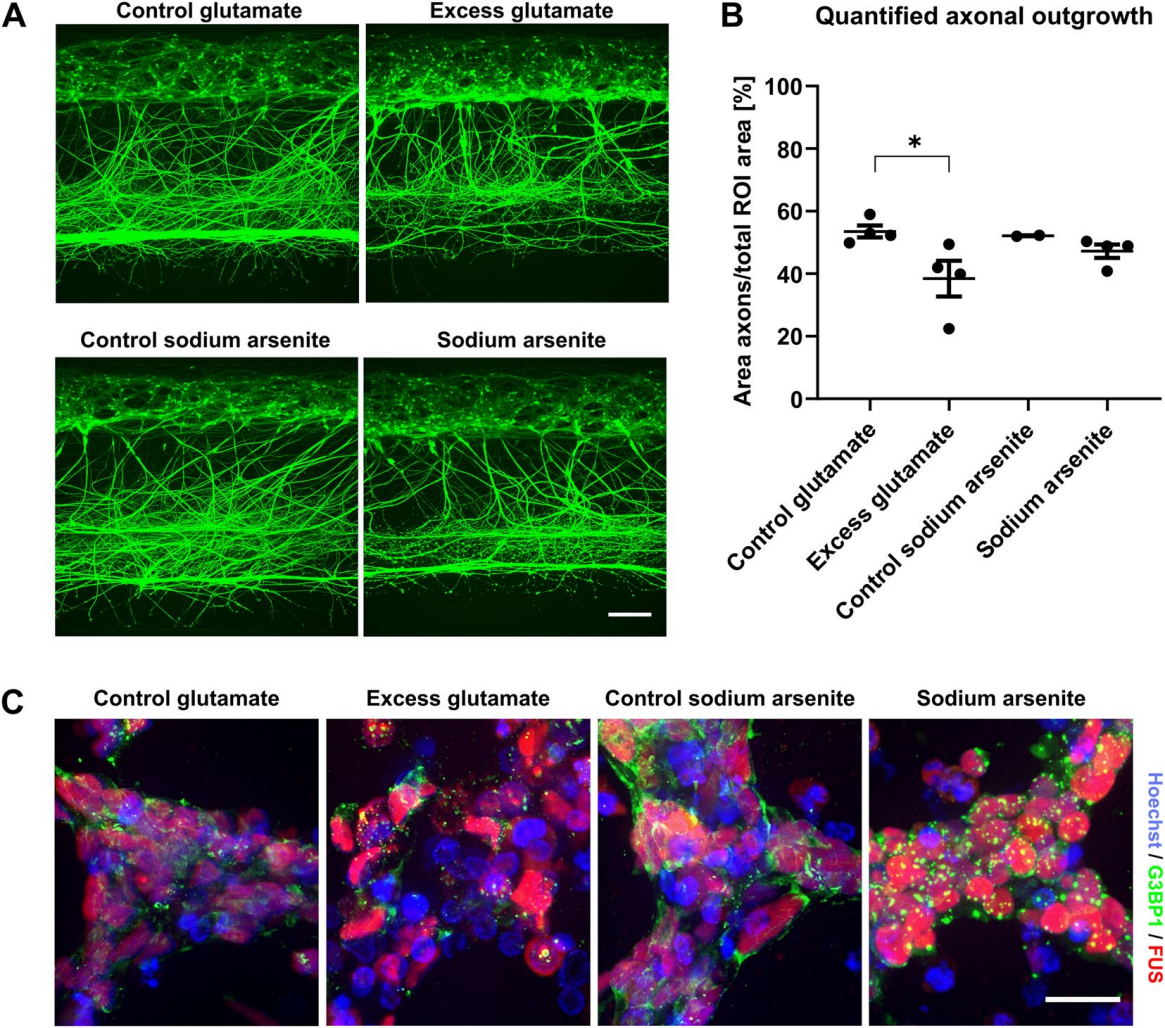
Excitotoxic conditions induce reduced axon outgrowth and formation of stress granules. (**A**) Motor neurons (day 17) were exposed to 5 mM glutamate for 7 days or 0.05 mM sodium arsenite for 3 h and labeled with calcein-AM dye. The scale bar is 200 µm. (**B**) Quantification of the axon outgrowth depicted in (**A**). Graph shows mean ± SEM. n = 2–4, *(P < 0.05); Kruskall-Wallis test. The graphic representation was created using GraphPad Prism, version 8.3.1 (https://www.graphpad.com/scientific-software/prism/). (**C**) Motor neurons (day 17) grown in an OrganoPlate 2-lane were exposed to 5 mM glutamate for 7 days or 0.05 mM sodium arsenite for 3 h. Cells were immunostained for the stress granule marker G3BP1 (green) and for FUS (red). Maximum projection images, the scale bar is 20 µm.

Glutamate toxicity is a hallmark of diseases such as ALS (Taylor et al., 2016). To examine whether we could model this pathological situation in the OrganoPlate culture, we exposed cultures to 5 mM glutamate for 7 days (day 10–17), in line with a previous study^32^. Excess glutamate significantly reduced axon outgrowth as compared to control treatment (P = 0.041; Fig. 5A,B). Exposure to sodium arsenite is another commonly used treatment for studying neuronal vulnerability in ALS and other diseases, as patient cells may respond differently to cellular stress^37–39^. Short-term exposure to sodium arsenite (3 h) did not significantly reduce axon outgrowth (Fig. 5A,B), but did induce formation of SGs, as did treatment with excess glutamate (Fig. 5C). SGs are accumulations of proteins and RNAs, mostly consisting of stalled translation initiation complexes, that form in a reversible manner upon cellular stress. Treatment with glutamate or sodium arsenite induced SG formation as shown by G3BP1 immunostainings. G3BP1 is a major component of SGs and shows a diffuse expression pattern in control conditions. Following both treatments, more granular patterns were found that are indicative of SG formation (Fig. 5C). We confirmed that the SGs are formed in neurons rather than non-neuronal cells, as results from immunostaining (data not shown) and qPCR analysis only showed sporadic presence of glial cells in our cultures. The low mRNA levels of s100β are presented in Supplementary Fig. S1. As expected, FUS remained mostly nuclear in all conditions (Fig. 5C), indicating that the incorporation of FUS in SGs requires ALS mutations in the *FUS* gene or other additional insults. Overall, these data show that the OrganoPlate motor neuron cultures can be used to study disease-relevant phenotypes, such as axon degeneration and SG formation.

### Axon outgrowth can be directed in the OrganoPlate

**Figure 6:**
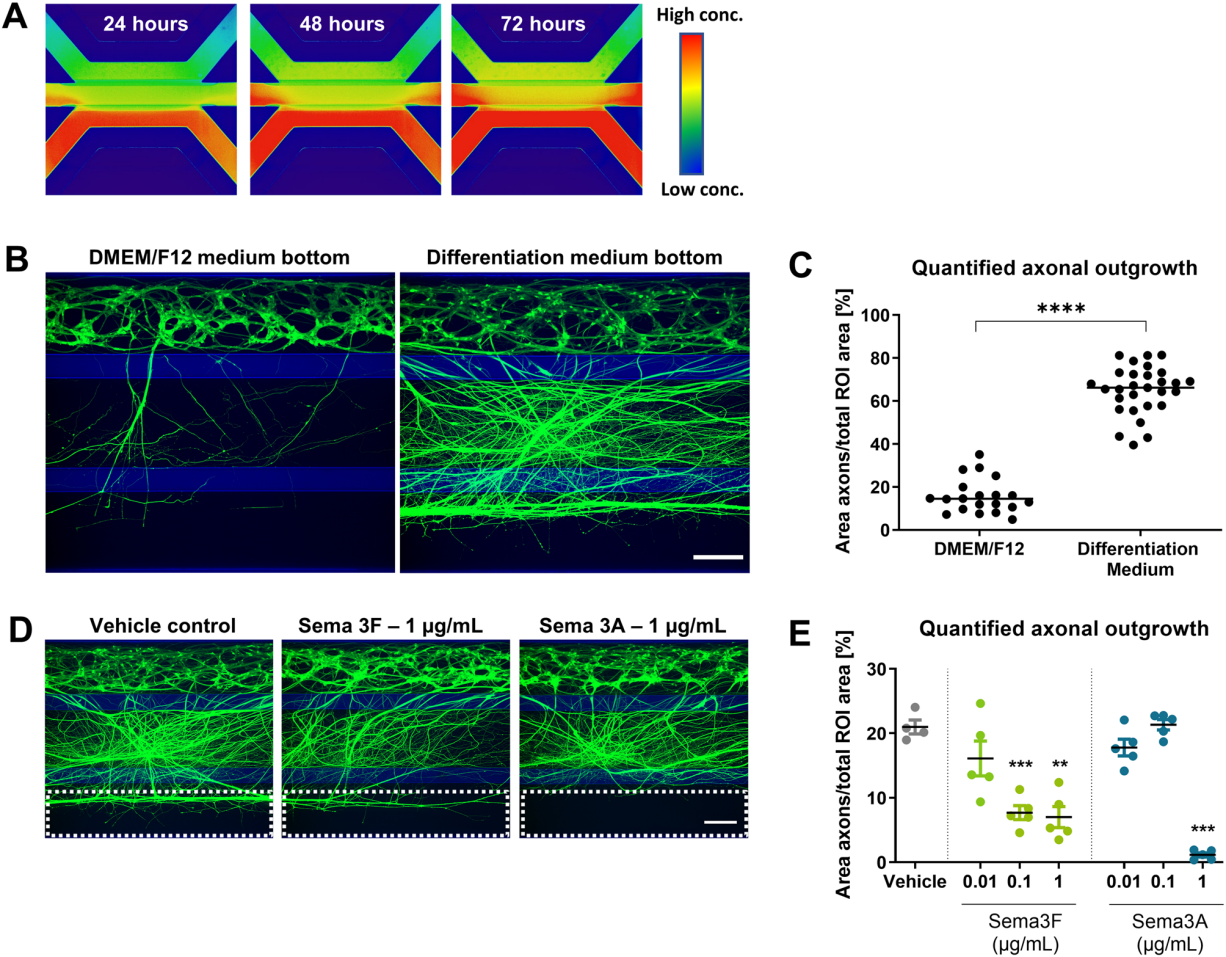
Attraction and repulsion of axons. (**A**) Gradient formation is visualized by perfusion of a fluorescein isocyanate (FITC)-Dextran (150 kDa) molecule through the bottom lane of a tissue culture chip. Images were taken at 24 h, 48 h, and 72 h after perfusion, showing stable gradient formation in the OrganoPlate. (**B**) Motor neurons (day 17) were cultured with either DMEM/F12 or motor neuron differentiation medium in the bottom lane to assess axon attraction. Cultures are visualized using calein-AM dye. Maximum projection images, the scale bar is 200 µm. (**C**) Quantification of axon outgrowth shown in (**B**) shows a significant (P < 0.0001) increase in outgrowth upon perfusion of the bottom lane with motor neuron differentiation medium. Graph shows mean ± SEM, n = 20–29, N = 2; unpaired t test. (**D**) Motor neurons (day 17) were cultured with either semaphorin 3F (SEMA3F) or 3A (SEMA3A) in the bottom lane to assess axon repulsion. Cultures are visualized using calein-AM dye. Maximum projection images, the scale bar is 200 µm. Dashed lines indicate the region of interest for axon outgrowth quantification in (**E**). (**E**) Quantification of the axon outgrowth depicted in (**D**). Axons were significantly less prevalent in the bottom microfluidic lane when perfused with SEMA3F (1 µg/mL, P = 0.001; 0.1 µg/mL, P = 0.0003) or SEMA3A (1 µg/mL, P = 0.001). Graph shows mean ± SEM, n = 5, *(P < 0.05), ** (P < 0.01), or *** (P < 0.001); Brown-Forsythe and Welch test. The graphic representations in (**B**) and (**D**) were created using GraphPad Prism, version 8.3.1 (https://www.graphpad.com/scientific-software/prism/).

The observation that axons extend towards the bottom lane, demonstrates that the OrganoPlate setup can be used for investigating directed human motor axonal outgrowth. This is of interest for studying motor axon biology or for devising regenerative strategies. Previously, gradient formation in the OrganoPlate has been reported to be stable for up to 6 days^40^. We now verify this for our assay setup. Figure 6A shows a gradient of 150 kDa fluorescein isothiocyanate (FITC)-Dextran molecules in our culture setup comprising neurons in matrigel. The gradient stays stable for at least 72 h. The stability of the gradients is longer than the time interval between medium changes (2–3 days), indicating that gradients can be maintained over the entire culture period (Fig. 6A).

We then attempted to use these gradients to direct axonal outgrowth. Interestingly, perfusion of the bottom lane with motor neuron differentiation medium caused significantly increased extension of axons into this lane as compared to perfusion with DMEM/F12 (P < 0.0001; Fig. 6B,C). In contrast, perfusion of the axonal repellents SEMA3A or SEMA3F^41–43^ in the bottom lane inhibited axonal outgrowth. The results show that SEMA3A (1 µg/mL) prevented axons from extending towards the edge of the gel region, as compared to the vehicle control (P = 0.0003; Fig. 6D,E). Application of SEMA3F induced a dose-dependent decrease in axons extending to the edge of the gel region, with concentrations of 1 µg/mL (P = 0.001) and 0.1 µg/mL (P = 0.0003) having statistically significant effects, when compared to the vehicle control. The edge of the gel extends into the lower perfusion region as a consequence of meniscus stretching during phaseguide-assisted patterning^44^. For this reason, quantification in Fig. 6C,E is based on a region of interest taken in the lower perfusion channel.

In conclusion, gradients formed in the OrganoPlate can be used to attract and repel motor axons. Moreover, gradient-based studies may also be extended to toxicity studies, by solely perfusing the bottom lane with the toxin. We demonstrated this with vincristine, where axons in the middle lane were again significantly affected using vincristine concentrations of 1 nM and higher (P < 0.0001, Supplementary Fig. S6). However, it is important to note that the steepness of the gradient may be altered due to the small molecular size of vincristine. Together, the results suggest that the OrganoPlate setup can be used for investigating directed human motor axon outgrowth, which is of interest for advancing our knowledge in motor axon biology or for devising regenerative strategies.

## Discussion

Here, we present a robust 3D model for studying human motor neurons and their axons in a perfused microfluidic platform. The platform enables segregated neurite outgrowth from motor neuron somata. Importantly, outgrowing neurites were demonstrated to be predominantly axonal. Outgrowth was shown to be highly reproducible and could be quantified by standard image analysis techniques. Moreover, axon outgrowth and stability were affected by a compound acting on the microtubule network. Furthermore, we could reproduce disease-relevant phenomena such as excitotoxicity and stress granule formation, while axons could also be attracted and repelled, demonstrating the ability to direct motor axonal outgrowth.

Segregation of axons from cell somata is of crucial importance for studying motor neuron diseases such as ALS. ALS is characterized by prominent axonal changes, eventually leading to axon degeneration and target de-innervation. Although changes such as altered axonal transport and cytoskeletal abnormalities have been reported^45,46^, early pathological changes in motor axons in ALS remain poorly characterized. However, they provide important therapeutic targets due to their early occurrence in the disease process. It is expected that high-throughput platforms can be used to identify reagents that target axon degeneration, which will accelerate drug development for ALS and other motor neuron diseases. To demonstrate the applicability of our model for compound testing, we exposed our cultures to a concentration range of vincristine. Vincristine is a chemotherapeutic agent which belongs to the vinca-alkaloids and primarily inhibits and disrupts microtubule networks by binding α- and β-tubulin monomers^35^. We show that vincristine caused a dose-dependent decrease in the axon density in the middle lane. Lack of clear changes in LDH activity indicates that axons may be affected in the absence of robust effects on cell viability in general. This observation not only confirms that changes in axon integrity can be modeled in our platform, but also that it is suitable for generating dose–response curves for specific compounds.

In our study, glutamate exposure led to a decrease in axon outgrowth and to a qualitative increase in the number of SGs. These results indicate that ALS-relevant phenotypes, such as glutamate toxicity and SG formation, can be captured in the OrganoPlate platform. It is thought that in ALS patients, motor neurons are exposed to pathological levels of extracellular glutamate, causing excitotoxicity^47^. Furthermore, motor neurons of most ALS patients show pathological protein aggregates that contain the RNA-binding protein TDP-43^48–50^. Other proteins such as the RNA binding protein FUS also localize in protein aggregates, but in a smaller group of patients^51,52^. Both TDP-43 and FUS play important roles in RNA processing and predominantly localize in the nucleus of healthy motor neurons. However, FUS and TDP-43 become more cytosolic in motor neurons of ALS patients, where they influence the assembly and dynamics of SGs^53^. SGs form when cells are exposed to adverse environmental conditions, such as heat shock, viral infection and oxidative stress^54^, and are believed to contribute to the formation of insoluble protein aggregates^53,55^. Changes in SG formation and dynamics are therefore thought to contribute to ALS pathology^37^. The mechanisms with which this happens may be studied more quantitatively in future studies.

The OrganoPlate may also be used to identify cues to enhance motor nerve regeneration following peripheral nerve or spinal cord injury. Motor axons often fail to successfully repair severed motor axon projections and no therapeutic interventions are available to stimulate this process. Since molecular gradients are known to influence axon outgrowth in vivo*,* we first demonstrated our ability to generate gradients in the OrganoPlate. We then attempted to utilize gradients to provide outgrowing axons with chemotrophic cues. Perfusion of motor neuron differentiation medium in the bottom lane increased axon outgrowth as compared to basal medium. This may either be explained by gradient formation of growth factors which influence the rate with which axons protrude into the middle lane, while it cannot be excluded that, upon perfusion of the bottom lane with basal medium, dilution of the differentiation medium contributes to the reduced axonal outgrowth.

Van Duinen et al.^40^ showed that a gradient could be maintained for many hours in the OrganoPlate. They demonstrated this is in a cell-free setup in which a gel was symmetrically perfused and in which one perfusion channel acted as a source and the other perfusion channel as a sink. Here we confirmed these results for our own assay setup. A crucial difference, however, is that our assay comprises neurons in matrigel that were inserted in the top lane. It should be expected that matrigel impedes perfusion flow in the top channel, thereby reducing the sink capacity. Our results show that the gradient is stable over prolonged periods, nonetheless. This may be explained by the interstitial flow through the matrigel, or by neuronal remodeling of the ECM which subsequently causes some level of perfusion flow.

We then perfused the bottom lane with semaphorins, which were expected to form directional repulsive cues. The molecular weight of these semaphorins upon dimerization (200–250 kDa) is higher than that of the FITC-dextran molecule (150 kDa) which has been used to confirm gradient formation. This implies that the semaphorin gradient is of similar or of lower steepness as compared to the gradient that we found for FITC-dextran. We prevented axons from protruding into the bottom microfluidic lane by providing SEMA3A or SEMA3F to this lane. Secreted class 3 semaphorins can act as axonal repellents via the Neuropilin receptor^41,43,56,57^. These results show that axons can be attracted and repelled in vitro in 3D in a segregated manner. Quantification was performed on the lower lane, where axons mostly bundle together at the interface of gel region with the perfusion lane. This causes some loss in spatial resolution as axons are overlapping in this region. We show, however, that this approach is still useful for capturing differences in axonal outgrowth. Such studies could employ the platform for studying the mechanism-of-action and function of various axon guidance cues as well as to search for reagents that could neutralize repulsive gradients, known to inhibit axon regeneration in vivo^58^, or to boost axon growth across sites of injury.

Evidently, motor axons do not grow in isolation but are surrounded by other cell types such as astrocytes and microglia in the central nervous system and Schwann cells in the peripheral nervous system, where they form synapses with skeletal muscle cells. The physiological relevance of our model can therefore be enhanced through the incorporation of these other cell types, which have shown to influence axon regeneration^59–61^, as well as ALS pathology^62–65^. The neuromuscular junction is of special interest, as it is affected at early stages in ALS^66^. In addition, the use of a chemically defined synthetic gel as an alternative to matrigel-GFR may be considered to further reduce the presence of growth factors in the ECM gel and to reduce possible batch-to-batch variation^67^.

The work described here employed motor neurons derived from a healthy donor and induced common motor neuron disease phenotypes by means of chemical triggers, such as excess glutamate and sodium arsenite. Although these techniques are widely used to study motor neuron diseases in vitro, a more complex approach can further improve the understanding of ALS disease mechanisms. Since diseases such as ALS are highly heterogeneous, it is likely that personalized treatments need to be developed for individual patients or specific patient groups. The compatibility of our platform with stem cell technology is beneficial for developing such personalized medicine approaches, allowing patient-derived material to be used^68,69^. Using the 3D neurite outgrowth model presented here, one can study differences between healthy and patient-derived motor neurons and their response to insults in a high-throughput ECM-embedded setting. Neurite outgrowth can be analyzed in 2D maximum projection images to assess high-level differences between conditions, as was done in this study, or in 3D at higher magnification to study individual somas and axons. Finally, the model may allow the development of other axon-based read-outs in the OrganoPlate. For example, axonal transport is widely disturbed in ALS and could be studied in 3D in our model^14,45,70^. As axonal pathology is not limited to motor neuron disease^71–73^, it will be interesting to assess whether growth of other neuronal populations in the OrganoPlate also results in axonal enrichment.

An important feature of the assay described here is that neurite outgrowth is 3D and not guided by mechanical cues such as microgrooves. In addition, particular attention was paid to the robustness and reproducibility of the assay, to allow smooth adoption by other laboratories. A crucial aspect is that all ingredients necessary for the assay are commercially available. The throughput of 40 chips per OrganoPlate, as well as its compatibility with standard microscopy and automation equipment, provides a good basis for routine experimentation. We thus foresee that the assay will be picked up by a wide audience.

In conclusion, we have established a motor neuron model with directed and segregated axon outgrowth, which can be used to study motor axons in 3D. We demonstrate the versatility of the model by showing changes in axon outgrowth upon exposure to various neurotoxins and signaling molecules. The platform will allow us to obtain further insight into motor axon biology and disease.

## Materials and methods

### Cell culture

iPSC-derived motor neuron progenitors (MNPs) (Axol, ax0078) were expanded in T75 flasks (734-2705, Corning, NY, USA) coated with 1:100 matrigel-GFR (Corning, 356341). MNPs were cultured in a chemically defined motor neuron progenitor expansion medium previously described by Du et al.^74^. The medium consists of equal parts DMEM/F12 with Glutamax (Gibco, 31331-028) and Neurobasal (Gibco, 21103-049) medium, supplemented with 0.5 × N2 supplement (Thermo Fisher, 17502-048), 0.5X B27 supplement (Thermo Fisher, 12587-010), 1% penicillin/streptomycin (Sigma, P4333), 0.1 mM ascorbic acid (Sigma, 49752), 3 µM CHIR99021 (Axon Medchem, 1386), 2 µM DMH-1 (Sigma, D8946), 2 µM SB431542 (Sigma, S4317), 0.5 µM purmorphamine (Enzo Life Sciences, ALX-420-045-M001), 0.5 mM VPA (Sigma, P4543-10G), and 0.1 µM retinoic acid (Sigma, R2625). Medium was refreshed three times a week. Cells were cultured up to passage 10 in a humidified incubator at 37 °C and 5% CO_2_ before seeding in the OrganoPlate. Cell detachment was performed by washing the cells 1 × using Phosphate Buffered Saline (PBS), after which cells were incubated for 5 min using accutase (Sigma, A6964).

### Culture of iPSC-derived MNPs in the OrganoPlate

iPSC-derived MNPs were seeded in an OrganoPlate 3-lane (MIMETAS, 4004-400B) for neurite outgrowth experiments. Each tissue culture chip consists of three microfluidic lanes of 400 µm × 220 µm (w × h) each^44^. Before seeding the MNPs, matrigel-GFR was thawed for a minimum of 3 h on ice. On the day of seeding, 50 µL of PBS was added to the observation windows to prevent the ECM from drying out and to increase optical clarity. Then, 1.1–1.4 µL of matrigel-GFR was dispensed into each middle inlet and the plate was incubated for 3.5 min at 37 °C, 5% CO_2,_ and 11.5 more at room temperature (RT). Alternatively, 1.7 µL of a collagen-I based ECM was disposed into the middle inlet, as previously described^44^, but with a 15-min incubation time. MNPs were then resuspended to a cell density of 30,000 cells/µL using matrigel-GFR, and 2.0 µL of this cell suspension was added to the top inlet. The plate was then again transferred to an incubator (37 °C, 5% CO_2_) for 15 min, after which a motor neuron differentiation medium was added to the top and bottom in- and outlet (50 µL to each well). The motor neuron differentiation medium used in this study has previously been described^74^ and consists of equal parts DMEM/F12 and Neurobasal medium, supplemented with 0.5 × N_2_ supplement, 0.5× B27 supplement, 1% penicillin/streptomycin, 0.1 mM ascorbic acid, 0.5 µM purmorphamine, and 0.1 µM retinoic acid. After day 6 of differentiation, 0.1 µM Compound E (Bio-Connect, AG-CR1-0081-C250) was added to the differentiation medium for the remaining culture time. The plate was then transferred to an incubator and placed on a rocking platform (7° inclination, 8-min interval), to initiate bidirectional perfusion flow.

For calcium imaging experiments and for the immunostainings following glutamate and sodium arsenite exposure, cells were seeded in an OrganoPlate 2-lane (MIMETAS, 9605-400B) with 200 µm × 200 µm (w × h) microfluidic lanes. Cells were detached and pelleted as described above. The OrganoPlate 2-lane was subsequently seeded as described previously^75^. Medium was added to the gel inlet, perfusion inlet and the perfusion outlet (50 µL to each well).

All read-outs were conducted on day 17, as the differentiation method used in this study was previously reported to yield mature motor neurons after 16 days of differentiation^74^. At day 17, the cultures in this study showed abundant and robust neurite outgrowth that could be quantified.

### Assessment of gradient formation and stability

Directly following MNP seeding to an OrganoPlate 3-lane, motor neuron differentiation medium supplemented with 0.5 mg/mL 150 kDa FITC-Dextran (Sigma, 48946) was perfused through the bottom lane (50 µL per inlet and outlet). Identical volumes of motor neuron differentiation medium without dye were again added to the top inlet and outlet. Images of the corresponding wavelength were then taken using an ImageXpress Micro XLS microscope (Molecular Devices, Sunnyvale, CA, USA) using a 4 × objective. The plate was placed back onto the rocking platform following each imaging session. Pictures were taken after 24, 48 and 72 h to assess the stability of the gradient.

### Calcein labeling of live motor neurons

To visualize live cells, including neurites, the medium was supplemented with 0.5 µg/mL calcein-AM (Thermo Fisher, C3099). Here, 50 µL of calcein-AM-supplemented medium was added to the top in- and outlets, while 25 µL was added to all middle and bottom in- and outlets. The plate was then placed back onto the rocking platform for 3 h. Images were captured using an ImageXpress Micro XLS-c microscope. Using a 10 × objective, Z-stacks with 3 µm spacing were captured and a maximum projection was saved per tissue culture chip. 3D reconstructions were rendered from a confocal Z-stack using Imaris Viewer (version: Viewer, Bitplane, Zürich, Switzerland, https://imaris.oxinst.com/imaris-viewer). ImageJ^76^ was used to depict the height of the cells in the Z-stack, rendering a 2D image.

### Neurite outgrowth quantification

Maximum projections were processed in ImageJ^76^ to quantify neurite outgrowth. The center of the middle lane of the chips was selected as the region of interest for the data shown in Figs. 1, 2, 3, 4 and 5, as this lane features the 3D axonal outgrowth of the model. In short, calcein signal from the middle lane was isolated and subsequently converted into a binary image using the Huang threshold ImageJ plugin. The binary image was then skeletonized using the skeletonization ImageJ plugin, after which the total neurite length could be extracted from the skeletonized image. For more complex neurite networks, we used a slightly different quantification approach. Here, a binary image was generated in an identical manner. The proportion of the binary image containing signal (and thus neurites) was calculated. After Fig. 2, neurite outgrowth is referred to as axonal outgrowth. Extracted values were then compared across conditions and these were plotted in GraphPad Prism, version 8.3.1 (GraphPad Software, San Diego, CA, USA, https://www.graphpad.com/scientific-software/prism/). The skeletonization approach was used in Fig. 2 and Supplementary Fig. S3 only.

### RNA isolation, cDNA synthesis and qPCR and data analysis

2D cultures of proliferating MNPs (Axol) and OrganoPlate cultures of 17-day differentiated motor neurons (from MNPs, Axol) were lysed using TRIzol (Thermo Fisher, 15596026). For the differentiated neurons, all contents of 40 OrganoPlate chips were lysed and pooled into one sample. Because the iPSCs from the same donor were not commercially available, iPSCs from a different donor were used as a reference and have previously been characterized more extensively, annotated as iPSC1^77,78^. Samples were stored at − 20 °C before RNA extraction. RNA was extracted and cDNA prepared as previously described^79^. Primers were intron-spanning and designed with PrimerBLAST (NCBI). The expression fold changes were determined (2^−ΔΔCT^) and normalized with the geomean of the three reference genes *GAPDH*, *BETA-ACTIN* and *TBPQ* (see primer sequences in Table 1). Graphical representations were created in GraphPad Prism, version 8.3.1 (GraphPad Software, San Diego, CA, USA, https://www.graphpad.com/scientific-software/prism/).

**Table 1:** qPCR primers used in this study.

Gene	5′-Forward primer-3′	5′-Reverse primer-3′
*BETA-ACTIN*	GTGGACATCCGCAAAGACC	TCTGCATCCTGTCGGCAAT
*CHAT*	GACGTCTGACGGGAGGAG	TCAATCATGTCCAGCGAGTC
*GAPDH*	TGTTCGACAGTCAGCCGCATCTTC	CAGAGTTAAAAGCAGCCCTGGTGA
*GFAP*	AGGTCCATGTGGAGCTTGAC	GCCATTGCCTCATACTGCGT
*ISL1*	AAGGACAAGAAGCGAAGCAT	TTCCTGTCATCCCCTGGATA
*MAP2*	TGCCTCAGAACAGACTGTCA	GGCTCTTGGTTACTCCGTCA
*NANOG*	GCCTGTGATTTGTGGGCCTGA	GTGGAAGAATCAGGGCTGTCCTG
*RBFOX3*	GACGCAATGGTTCAGCCTTTT	GCGTACTTCCGTAGAGTGTCAG
*OLIG2*	CGC ATA GCG TCT GTG TTC A	CAC TGC CTC CTA GCT TGT CC
*PAX6*	AGTTCTTCGCAACCTGGCTA	ATTCTCTCCCCCTCCTTCCT
*S100BETA*	TGGAAAAAGCAACTCCATCAGAA	GAATCGCATGGGTCACGG
*NFH*	GCAGTCCGAGGAGTGGTTC	CGCATAGCGTCTGTGTTCA
*SOX2*	CGAGGGAAATGGGAGGGGTGC	TGCAGCTGTCATTTGCTGTGGGT
*SYNAPSIN*	AGTTCTTCGGAATGGGGTGAA	CAA ACT GCG GTA GTC TCC GTT
*TBPQ*	CCACAGCTCTTCCACTCACA	GCGGTACAATCCCAGAACTC
*VACHT*	CTGCTAGTGAACCCCTTGAGC	CAGGACTGTAGAGGCGAACAT

**Table 2:** Antibodies and buffers used in this study.

Antibody	Company	Catalog #	Dilution	Serum
TAU	Merck Milipore	05-803	1:500	Buffer 1
MAP2	Neuromics	CH22013	1:2000	Buffer 1
ISL1	DSHB	30.2D6	1:400	Buffer 1
CHAT	Merck Milipore	AB144P	1:50	Buffer 2
Neurofilament H (clone SMI-32)	Biologend	801702	1:1000	Buffer 1
G3BP1	Abcam	ab56574	1:500	
FUS	Novus Biologicals	NB100-2599	1:100	Buffer 1
Alexa Fluor goat-anti-mouse 488	Life Technologies	A32723	1:250	Buffer 1
Alexa Fluor goat-anti-rabbit 488	Life Technologies	A32731	1:250	Buffer 1
Alexa Fluor donkey-anti-goat 488	Thermo Fisher	A32814	1:250	Buffer 2
Alexa Fluor goat-anti-chicken 647	Abcam	Ab150171	1:500	Buffer 1
Donkey anti-rabbit CF 647	Sigma	SAB4600177	1:250	Buffer 1

Chemical	Company	Catalog #	Dilution buffer 1	Dilution buffer 2
Triton X-100	Sigma	T8787	1%	1%
Bovine serum albumin	Sigma	P6148	2%	2%
Normal donkey serum	Abcam	Ab7475		1%
Goat serum	Sigma	G9023	5%	

### Immunocytochemistry

At day 17 following MNP seeding, cultures were fixed using 4% paraformaldehyde (PFA; Sigma, P6148) in PBS. The following steps were performed as described previously^44^, with minor modifications. Cells were washed with PBS, blocked using 2% BSA, 0.1% Triton X-100 and either 5% goat serum or 1% normal donkey serum (NDS) when staining for CHAT. Moreover, 1% Triton X-100 was used in the blocking buffers for G3BP1 and FUS stainings only. Antibodies were diluted in a blocking buffer containing 2% BSA, 1% Triton X-100 and 1% NDS or 5% goat serum and incubated for 2–3 day and DNA was labeled using Hoechst (Thermo Fisher, H3570). Images were subsequently captured with an ImageXpress Micro XLS microscope. Using a 10 × objective, Z-stacks were captured with 3 µm spacing and a maximum projection was saved per tissue culture chip. For Fig. 5, images were captured using a 40 × objective. Immunostainings were processed using ImageJ^76^. Immunostainings for ISL1 and CHAT, MAP2 and TAU were despeckled (ImageJ plugin) during analysis to remove background signal. All antibodies, buffers, and antibody dilutions are listed in Table 2.

### Calcium imaging

Cells cultured in an OrganoPlate 2-lane were incubated with Cal-520 AM to measure spontaneous calcium fluctuations. Motor neuron differentiation medium was supplemented with 200 µM Cal-520 AM and 1:250 pluronic F-120 (Thermo Fisher, P3000MP). 30 µL of solution was added to the medium inlet and 20 µL to the medium outlet. Cells were incubated under low perfusion (2°, 8 min) in 37 °C, 5% CO_2_ conditions for 90 min, followed by 30 min at RT in the dark. Images were captured at 2.08 Hz using an ImageXpress Micro XLS microscope (4 × magnification) and were subsequently processed in ImageJ to allow the visualization of the calcium fluctuations. Calcium recordings were corrected for bleaching (exponential fit) using a bleach correction plugin in ImageJ^76^. Recordings depict changes in intracellular calcium concentrations over time. Red depicts large changes in intracellular fluorescence and blue depicts small changes.

### Vincristine exposure

MNPs cultured in the OrganoPlate 3-lane were exposed to vincristine (Sigma, V8879) from day 13 to day 17. A medium change was performed on day 15. On day 17, a calcein labeling experiment was performed while the old medium was sampled to assess cell viability using a lactate dehydrogenase (LDH) activity assay. The results for the axonal outgrowth quantification were plotted in a dose–response curve. Here, the ROI involved the bottom lane. The dotted lines interconnected the 95% confidence intervals for each concentration. The graph was created in GraphPad Prism, version 8.3.1 (GraphPad Software, San Diego, CA, USA, https://www.graphpad.com/scientific-software/prism/).

### LDH activity assay

Lactate dehydrogenase (LDH) activity was analyzed using an LDH activity kit (Sigma, MAK066). LDH reduces NAD to NADH, which can be detected in a spectrophotometric multiwell plate reader**.** The assay was performed according to manufacturer’s protocol and has been previously described^80^. In short, at day 17, medium from the top microfluidic lane was sampled, and used for the LDH activity assay. Measurements were done after 1 min and every 2 min following the initial measurement. Absorbance was measured at 450 nm for a total duration of 50 min.

### Excitotoxicity assay in the OrganoPlate

MNPs were exposed to high glutamate from day 10 to 17. Glutamic acid (Sigma, G1251) was freshly dissolved in motor neuron differentiation medium to a concentration of 5 mM before performing a medium change. On day 17, cells were exposed to 0.05 mM sodium arsenite (Merck Milipore, 1062771000) for 3 h as a positive control for SG formation. Following exposure, MNPs in the OrganoPlate 3-lane were stained using calcein-AM, while the OrganoPlate 2-lane was fixated using 4% paraformaldehyde.

### Semaphorin exposure

Directly following seeding, motor neuron differentiation medium was supplemented with 0, 10, 100 or 1000 µg/mL semaphorin 3A (SEMA3A, Peprotech, 15017H) or semaphorin 3F (SEMA3F, R&D, 9878S3025) and perfused throughout the bottom lane (50 µL/well). Regular differentiation medium was added to the top lane (50 µL/well). Medium was changed every 2–3 days and calcein labeling was performed at day 17. Axonal outgrowth was quantified using a similar approach as described earlier, with the exception that the outgrowth in the bottom lane was quantified.

### Statistical design and statistical analyses

Data was analyzed using GraphPad Prism, version 8.3.1 (GraphPad Software, San Diego, CA, USA, https://www.graphpad.com/scientific-software/prism/). Data normality was assessed using the Kolmogoriv-Smirnov, Shapiro–Wilk, D’Agostino–Pearson omnibus and the Anderson–Darling tests. Equality of variances was assessed using the Brown–Forsythe and Bartlett’s tests, or using the F-test when performing a t-test. In case the assumptions were not violated, unpaired t-tests or one-way ANOVAs were performed. For one-way ANOVAs, Tukey’s multiple comparisons tests were performed. In case the assumption of equality of variances was violated, the Brown-Forsythe and Welch test was performed with a Dunnett T3 multiple comparisons test. When data was not normally distributed, the nonparametric Kruskall–Willis with Dunn’s multiple comparisons test was performed. For multiple comparisons tests, all groups were compared with all groups, unless stated otherwise. Statistical significance is indicated by one or more asterisks. *(P < 0.05), **(P < 0.01), ***(P < 0.001), or ****(P < 0.0001). Following multiple comparisons tests, p-values adjusted for these comparisons are reported. The total number of technical replicates is depicted as n, while the number of independent experiments is depicted as N.

## Supplementary Information

Supplementary Video 1.

Supplementary Video 2.

Supplementary Information.

## Data Availability

The datasets generated during in this study are available from the corresponding authors on reasonable request.
