# Supplementary figures and images for: A directional 3D neurite outgrowth model for studying motor axon biology and disease
Source: Sci Rep. 2021 Jan 22;11:2080. doi: 10.1038/s41598-021-81335-z (PMC7822896; doi:10.1038/s41598-021-81335-z)

## Slide 1
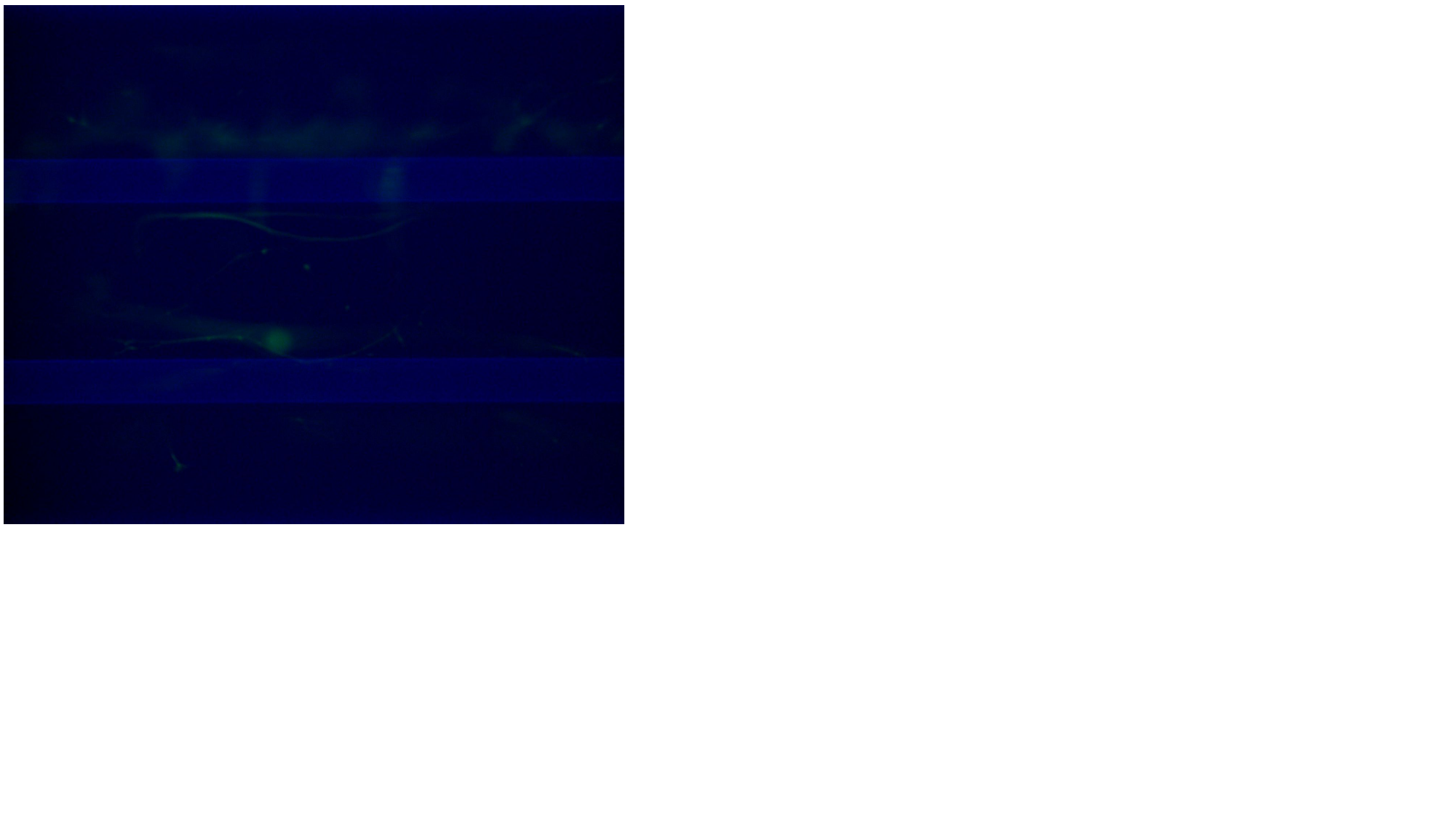

Supplement: Supplementary file 1 — Supplementary Video 1. [file 41598_2021_81335_MOESM1_ESM.pptx]

## Slide 1
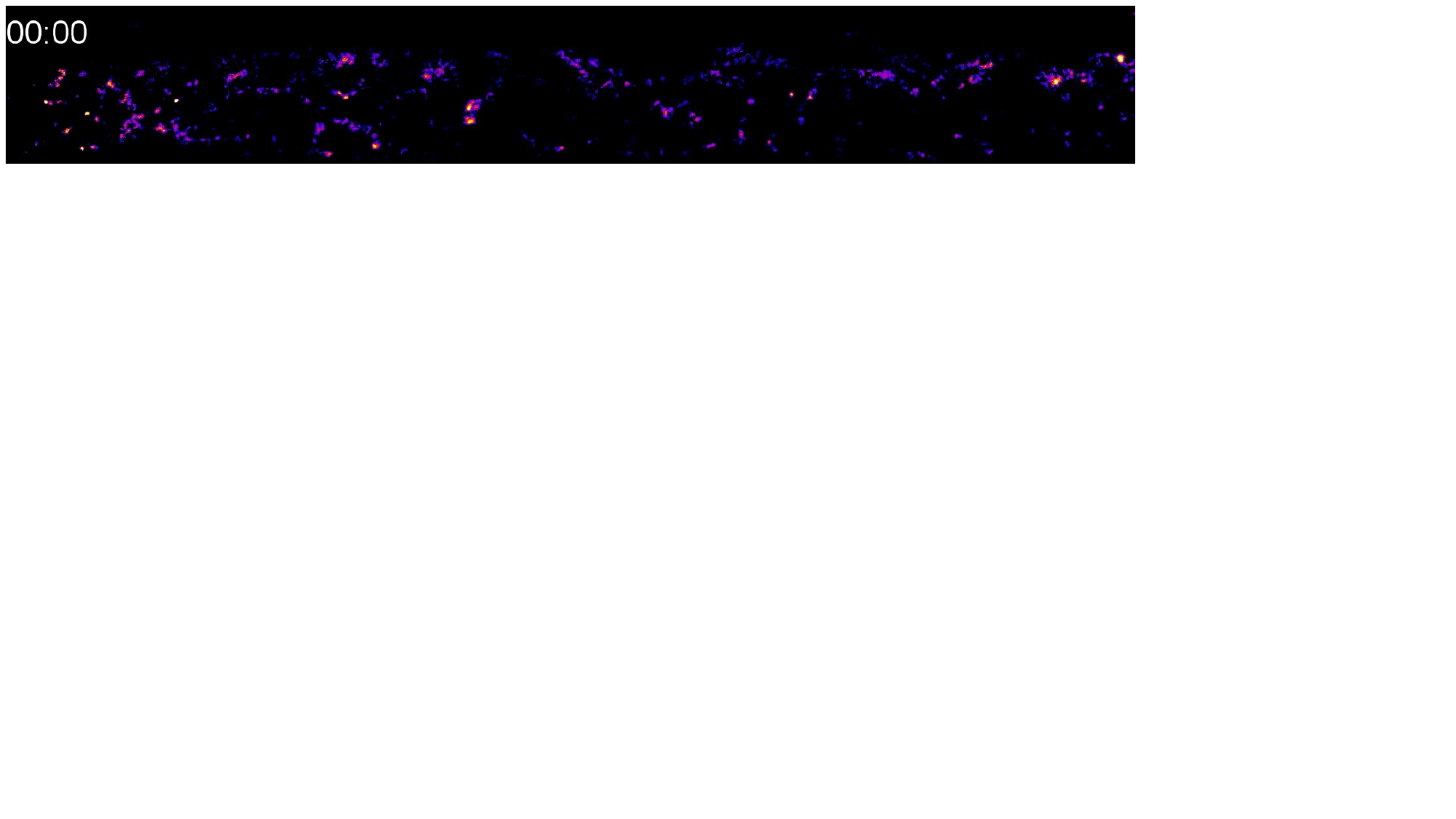

Supplement: Supplementary file 2 — Supplementary Video 2. [file 41598_2021_81335_MOESM2_ESM.pptx]
