# Supplementary material for: A directional 3D neurite outgrowth model for studying motor axon biology and disease
Source: Sci Rep. 2021 Jan 22;11:2080. doi: 10.1038/s41598-021-81335-z (PMC7822896; doi:10.1038/s41598-021-81335-z)
Supplement: Supplementary file 3 — Supplementary Information. [file 41598_2021_81335_MOESM3_ESM.pdf]

Supplementary information

A directional 3D neurite outgrowth model for studying motor axon biology and disease

Xandor M. Spijkers[1,2], Svetlana Pasteuning-Vuhman[2], Jennifa C. Dorleijn[1], Paul Vulto[1*], Nienke R. Wevers[1,3], and R. Jeroen Pasterkamp[2*]

[1] Mimetas BV, Organ-on-a-chip Company, 2333 CH Leiden, The Netherlands.
[2] Department of Translational Neuroscience, University Medical Center Utrecht Brain Center, Utrecht University 3584 CG, Utrecht, The Netherlands.
[3] Department of Cell and Chemical Biology, Leiden University Medical Centre, Einthovenweg 20, 2333 ZC, Leiden, The Netherlands

* Correspondence:

Paul Vulto (p.vulto@mimetas.com)
Jeroen Pasterkamp (r.j.pasterkamp@umcutrecht.nl)

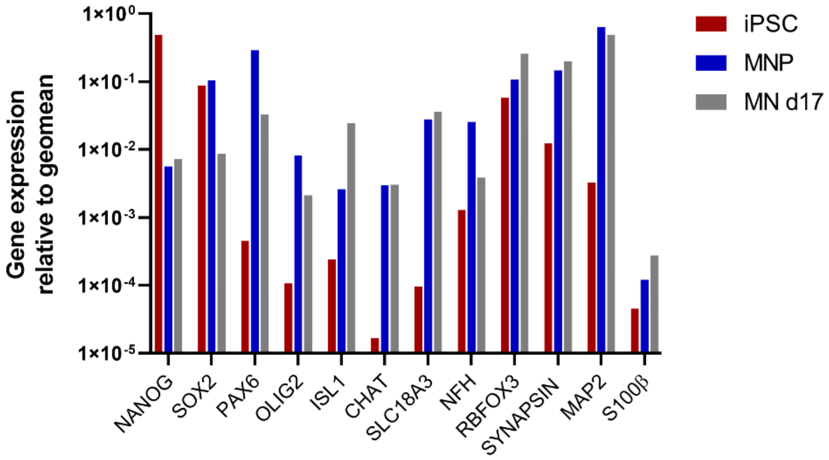

Supplementary Figure S1. Normalized gene expression levels. mRNA expression levels of induced pluripotent stem cells (iPSC), motor neuron progenitors (MNP), and motor neurons differentiated for 17 days (MN d17) in the OrganoPlate, normalized to the geomean which consists of the reference genes *GAPDH, BETA-ACTIN* and *TBPQ* to obtain the ΔCt values. These are then displayed as $2^{-\Delta Ct}$. The graphic representation was created using GraphPad Prism, version 8.3.1 (https://www.graphpad.com/scientific-software/prism/). RNA from MN d17 reflects one pooled sample of 40 OrganoPlate tissue culture chips, resulting in one biological replicate. qPCR experiments were performed in duplicate or triplicate (technical replicates).

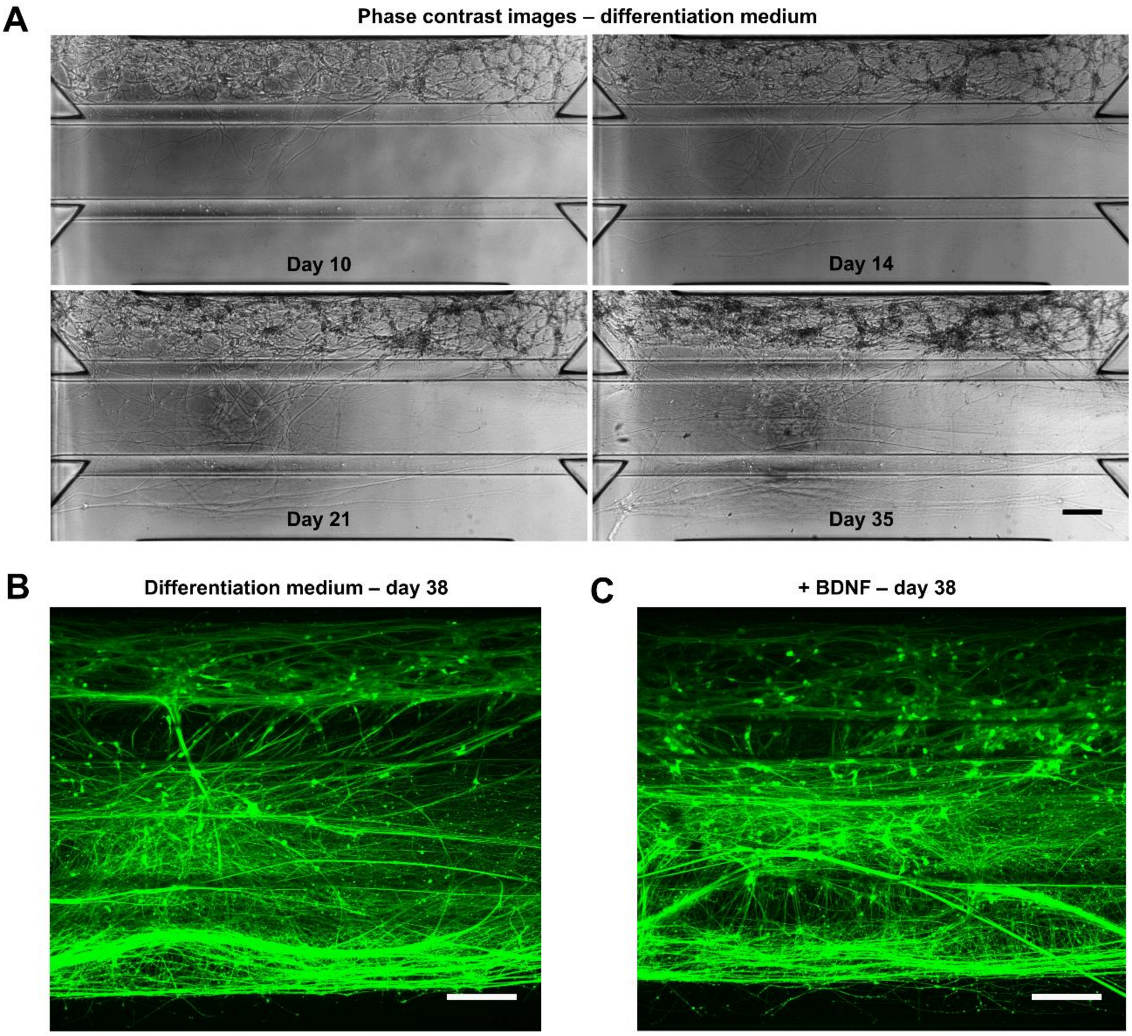

Supplementary Figure S2. Long-term culturing of 3D axonal networks and brain-derived neurotrophic factor affects somal migration. **(A)** Phase contrast images capturing axonal outgrowth over time, when the motor neuron differentiation medium is added to the top and bottom lane. **(B)** The complexity of the neuronal network at day 38 is visualized by loading live motor neurons with a calcein-AM dye. **(C)** The neuronal network at day 38 is visualized with a calcein-AM dye, where 10 ng/mL brain-derived neurotrophic factor (BDNF) was perfused throughout the bottom lane during the entire differentiation period. Scale bars are 200 µm.

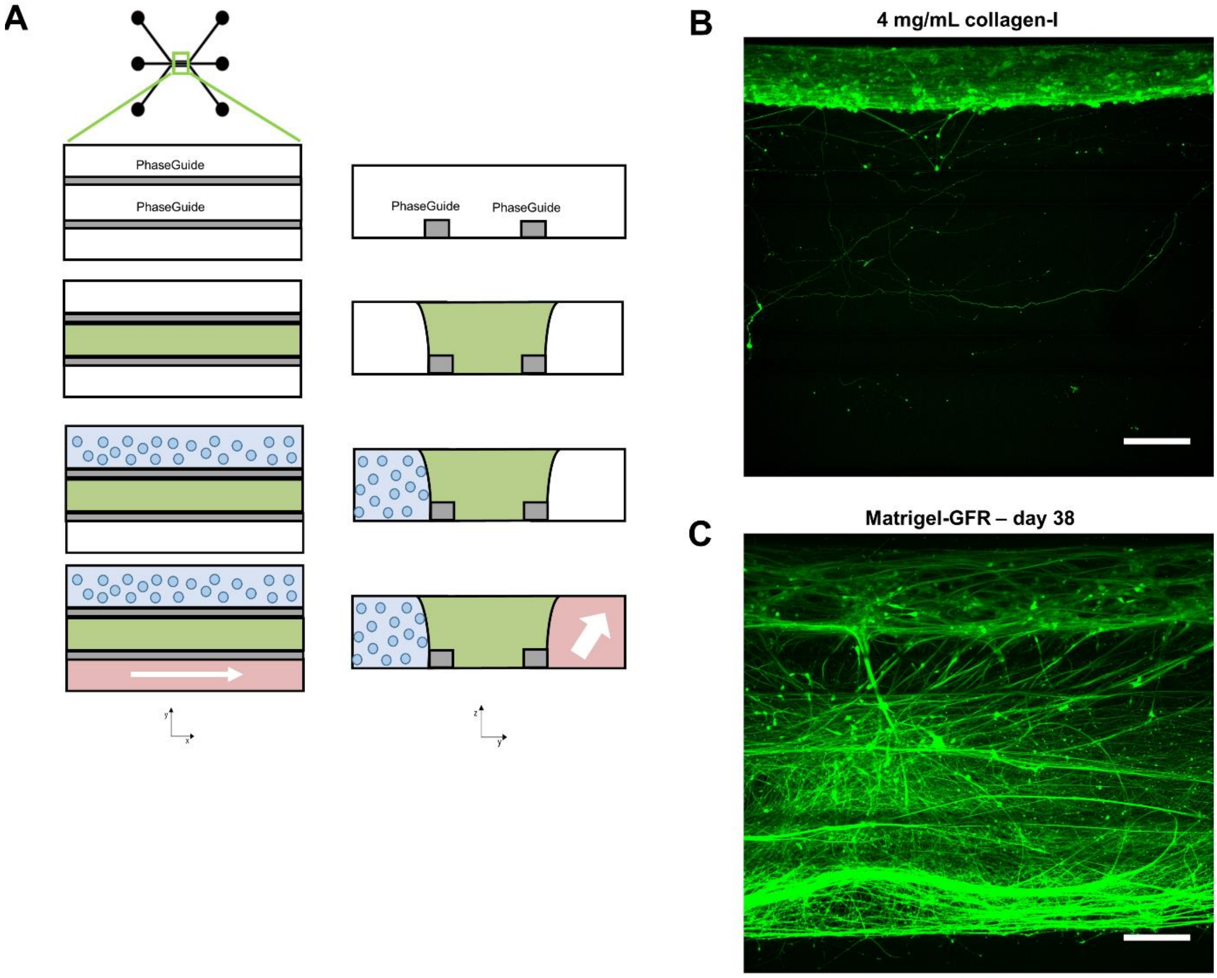

Supplementary Figure S3. Axonal outgrowth using a commercially available collagen-I matrix. **(A)** An overview of the seeding procedure using a commercially available collagen-I matrix is depicted. Collagen-I (4 mg/mL) was dispensed into the middle lane, after which MNPs embedded in matrigel-GFR were seeded into the top lane. Motor neuron differentiation medium was added to the top and bottom lane after which perfusion was initiated. The image is copyright of MIMETAS BV. **(B)** Motor neurons were labelled with calcein at day 35, where the middle lane contains collagen-I. **(C)** Motor neurons were labelled with calcein at day 38, where the middle lane is filled with matrigel-GFR. Scale bars are 200 µm.

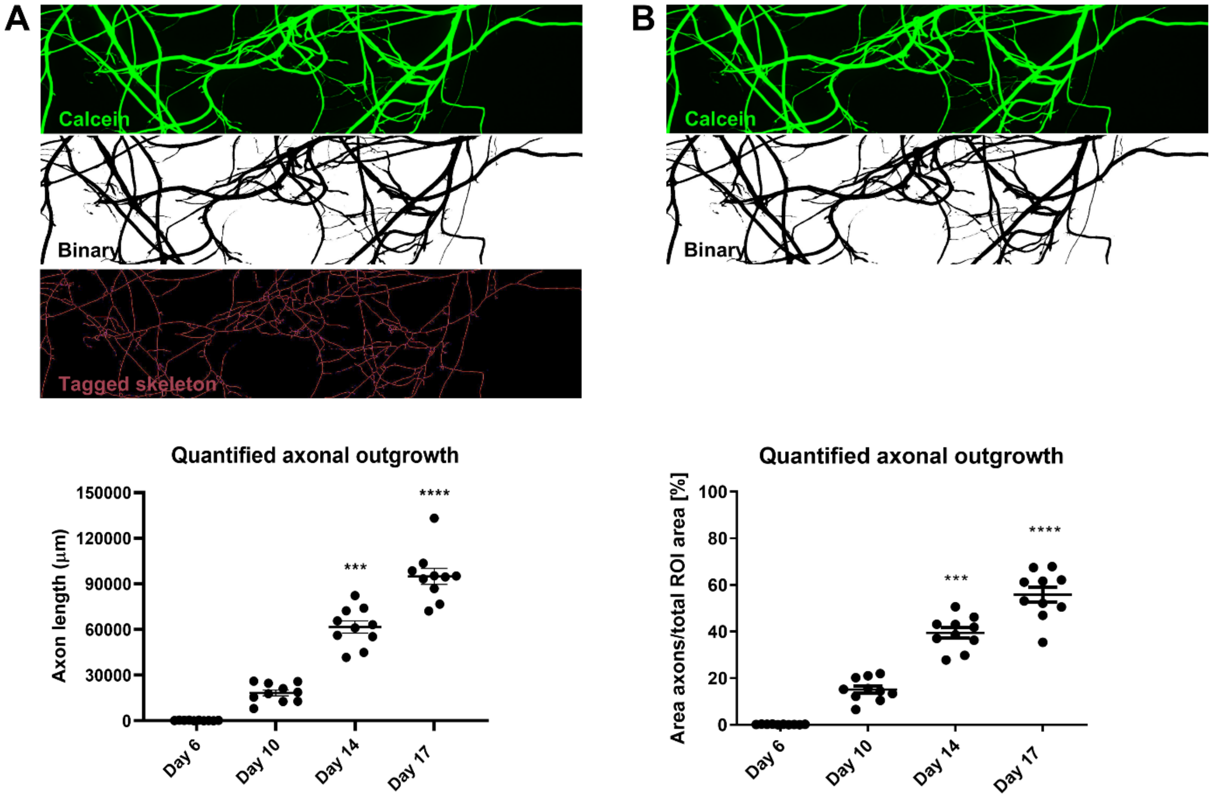

Supplementary Figure S4. Quantification of axonal outgrowth using two ImageJ-based approaches. **(A)** Quantification of axonal length in the ROI was done by first converting the raw data (calcein, green) into a binary image (black), which was then skeletonized (red). The length of the total skeleton was measured in µm. **(B)** An alternative approach to quantify axonal outgrowth is achieved by measuring the black signal of the binary image, as a ratio of the total ROI area. The results are expressed as mean values ± SEM of ten chips (n = 10 chips) per condition of two independent experiments (N=2), *(P < 0.05), ** (P < 0.01), *** (P < 0.001), or **** (P < 0.0001). The graphic representations were created using GraphPad Prism, version 8.3.1 (https://www.graphpad.com/scientific-software/prism/).

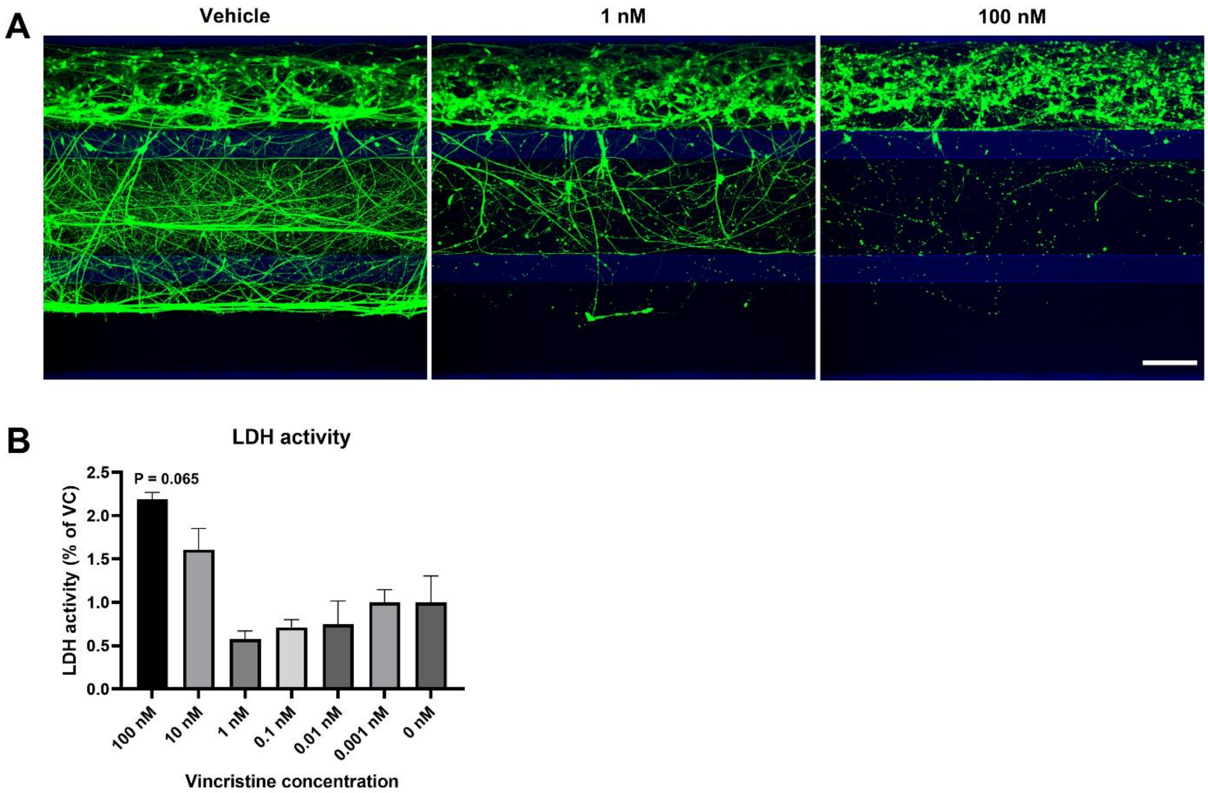

Supplementary Figure S5. Lactate dehydrogenase activity assay following vincristine exposure. **(A)** Calcein labelled motor neurons were exposed to to 0 nM, 1 nM, or 100 nM of vincristine for a duration of 4 days, showing dose-dependent damaging effects on axonal outgrowth and morphology. Scale bars are 200 µm. **(B)** LDH activity assay was conducted four days after vincristine exposure was initiated, medium was sampled and subjected to the assay. The 100 nM exposure resulted in a p-value close to the significance threshold when compared to the vehicle control (P = 0.065; Kruskall-Wallis test). The results are expressed as mean values ± SEM of five chips (n = 5 chips) per condition. The graphic representation was created using GraphPad Prism, version 8.3.1 (https://www.graphpad.com/scientific-software/prism/).

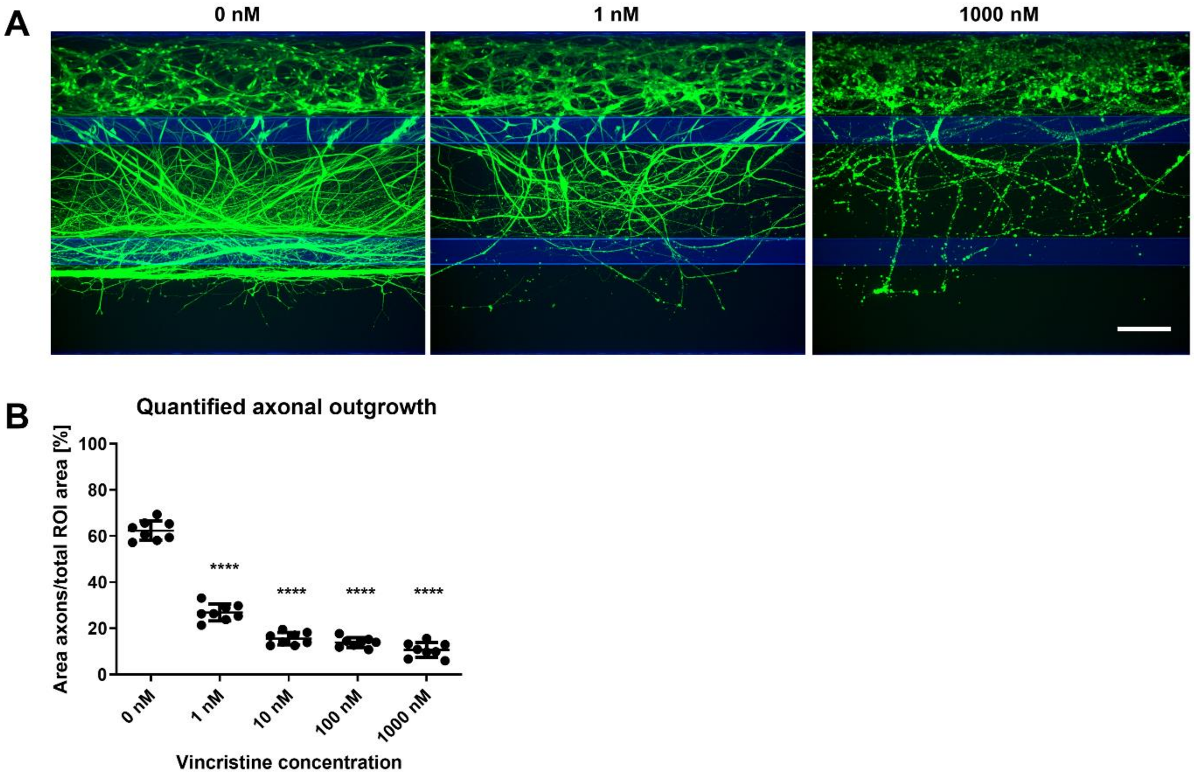

Supplementary Figure S6. Axonal outgrowth in the OrganoPlate® after vincristine exposure. **(A)** Calcein labelled motor neurons at day 17 are shown following a 4-day exposure to various vincristine concentrations (0 nM, 1 nM and 1000 nM). Scale bars are 200 µm. **(B)** Quantification of the axonal outgrowth revealed a dose-dependent decrease upon treatment with vincristine. The results are expressed as mean values ± SEM of eight chips (n = 8 chips) per condition, **** ($P < 0.0001$); one-way ANOVA. The graphic representation was created using GraphPad Prism, version 8.3.1 (https://www.graphpad.com/scientific-software/prism/).

Supplementary Video S1. Motor neurons display calcium fluctuations after 17 days of differentiation in the OrganoPlate. Motor neuron progenitors were embedded in matrigel-GFR in an OrganoPlate 2-lane and differentiated for 17 days before the cells were loaded with a calcium sensitive dye. Recording of intracellular calcium fluctuations are shown, where the color corresponds to the height of the difference in calcium signal. A red signal corresponds to a big difference in intracellular calcium concentrations, whereas a blue signal indicates a small difference.

Supplementary Video S2. A Z-stack demonstrating the 3-dimensionality of the cultures. At day 17, cells were labelled with calcein and a Z-stack consisting of 64 slices with 3 µm spacing was captured. The Z-stack is then shown as a video, where the first frame corresponds to the bottom of the Z-stack, and the last frame to the top of the Z-stack. The Z-stack shows specific cells coming into focus, depending on the height in the Z-stack thereby validating the 3D nature of the culture.
